# Low-Temperature Virus vB_EcoM_VR26 Shows Potential in Biocontrol of STEC O26:H11

**DOI:** 10.3390/foods10071500

**Published:** 2021-06-28

**Authors:** Aurelija Zajančkauskaitė, Algirdas Noreika, Rasa Rutkienė, Rolandas Meškys, Laura Kaliniene

**Affiliations:** Department of Molecular Microbiology and Biotechnology, Institute of Biochemistry, Life Sciences Center, Vilnius University, Sauletekio av. 7, LT-10257 Vilnius, Lithuania; aurelija.zajanckauskaite@bchi.vu.lt (A.Z.); algirdas.noreika@gmc.vu.lt (A.N.); rasa.rutkiene@bchi.vu.lt (R.R.); rolandas.meskys@bchi.vu.lt (R.M.)

**Keywords:** phage, biocontrol, VR26, STEC, O26:H11

## Abstract

Shiga toxin-producing *Escherichia coli* (STEC) O26:H11 is an emerging foodborne pathogen of growing concern. Since current strategies to control microbial contamination in foodstuffs do not guarantee the elimination of O26:H11, novel approaches are needed. Bacteriophages present an alternative to traditional biocontrol methods used in the food industry. Here, a previously isolated bacteriophage vB_EcoM_VR26 (VR26), adapted to grow at common refrigeration temperatures (4 and 8 °C), has been evaluated for its potential as a biocontrol agent against O26:H11. After 2 h of treatment in broth, VR26 reduced O26:H11 numbers (*p* < 0.01) by > 2 log_10_ at 22 °C, and ~3 log_10_ at 4 °C. No bacterial regrowth was observed after 24 h of treatment at both temperatures. When VR26 was introduced to O26:H11-inoculated lettuce, ~2.0 log_10_ CFU/piece reduction was observed at 4, 8, and 22 °C. No survivors were detected after 4 and 6 h at 8 and 4 °C, respectively. Although at 22 °C, bacterial regrowth was observed after 6 h of treatment, O26:H11 counts on non-treated samples were >2 log_10_ CFU/piece higher than on phage-treated ones (*p* < 0.02). This, and the ability of VR26 to survive over a pH range of 3–11, indicates that VR26 could be used to control STEC O26:H11 in the food industry.

## 1. Introduction

*Escherichia coli* (*E. coli*) is a gram-negative, facultatively anaerobic, rod-shaped bacterium of the genus *Escherichia*, family *Enterobacteriaceae*, that is commonly found in the gut of humans and warm-blooded animals [1]. Since the bacterium is routinely shed into the environment through feces, it can contaminate drinking water, irrigation water, and soil. Consequently, it can be transmitted to humans primarily through the consumption of contaminated foods [2,3]. Most strains of *E. coli* are non-pathogenic, however, a number of strains, such as Shiga toxin-producing *E. coli* (STEC), can cause severe foodborne disease. The Food and Agriculture Organization (FAO) together with the World Health Organization (WHO) estimated that more than 1.2 million new cases resulting in 128 deaths and nearly 13,000 Disability Adjusted Life Years (DALYs) occur each year because of contamination by the foodborne STEC [4]. Based on the results of outbreak surveillance data, the most frequently attributed sources of STEC globally were: beef (16%), produce (fruits and vegetables, especially leafy greens; 15%), and dairy products (6%) [3,4]. The association of STEC with produce presents a serious problem because these products are minimally processed. Moreover, STEC strains have been shown to be adapted to survive for prolonged periods on plants and are often resistant to washing and chemical sanitizers [5,6,7,8]. As a result, contamination of fresh produce is emerging as a major food safety challenge, suggesting that current strategies to control microbial population during the production of fresh produce do not guarantee the elimination of STEC [2,8,9,10,11,12]. Hence, novel approaches are urgently needed for controlling these foodborne pathogens.

A promising alternative in the food industry for the elimination of STEC from fresh produce is bacteriophages (phages): viruses that infect and kill bacteria [13]. There are many advantages of using phages in the biocontrol of foodborne pathogens. First, due to high specificity, bacteriophages target only specific bacteria without disturbing the normal microflora of foods. Second, while harmless to humans, phages are able to kill even multidrug-resistant bacteria. Third, phage preparations are considered the most environmentally-friendly antimicrobials since, usually, they contain only natural (genetically unmodified) phages in a weak saline solution [13]. In 2006 the US Food and Drug Administration (FDA) approved the use of a first bacteriophage preparation (LMP-102) to be used against *Listeria monocytogenes* on foods [14]. Since then, a number of phage products (such as ListShield™, EcoShield™, and SalmoFresh™ by Intralytix, and Listex™ P100 by Micreos) have been commercialized and have been approved for use in a growing number of countries [13,15]. Moreover, over the past 10 years, the number of research reports describing the use of phage biocontrol to target a variety of bacterial pathogens in various foods have increased dramatically [13,15]. With regard to STEC serotypes, however, most phage preparations, and therefore studies, have predominantly focused on O157:H7 [13,15,16,17].

The most common STEC serotype associated with human disease is, indeed, O157:H7 [2,4,18]. However, a growing number of foodborne human STEC infections are caused by non-O157 strains, among which O26:H11 is most frequently associated with severe diarrhea and hemolytic uremic syndrome worldwide [4,19,20,21,22]. Nevertheless, to the best of our knowledge, only a few O26:H11-targeting phages are described in the literature [23,24,25,26] and even fewer of those have been evaluated for their ability to reduce numbers of O26:H11 in a broth culture system, under standard laboratory conditions [27,28]. None of these phages, however, have been tested for their ability to control this particular pathogen on fresh produce.

Previously, we presented the complete genome sequence of a T4-related *E. coli* phage vB_EcoM_VR26 (VR26) and showed that none of the predicted VR26 proteins exhibited sequence homology with known integration-related proteins, antibiotic resistance determinants, or virulence factors. [29]. VR26 is a low-temperature virus, adapted to replicate at temperatures suboptimal for its host (<10 °C). The vast majority of characterized coliphages are mesophilic viruses, plating in the range of 15 to 42 °C [29,30]. To date, only seven *E. coli* phages, all belonging to the family *Myoviridae*, have been shown to be adapted to lyse their hosts at low temperatures (4–7 °C) [29,30]. Due to their unique physiological properties, such phages have tremendous potential in food safety applications, where lytic activity at low temperatures is especially needed. Therefore, the objective of this study was to evaluate the efficacy of VR26 in biocontrol of STEC O26:H11 both in vitro and on fresh produce.

## 2. Materials and Methods

### 2.1. Bacterial Strains and Culture Conditions

Bacterial strains used in this study are listed in Table 1. STEC strains, isolated from food samples, were kindly provided by Prof. Edita Sužiedėlienė (Institute of Biosciences, Life Sciences Center, Vilnius University, Vilnius, Lithuania), whereas *E. coli* MH1 was a gift from Prof. Kenneth N. Kreuzer [31]. For inoculum preparation, bacteria were streaked on LB (Luria-Bertani) agar and the plates were incubated at 37 °C for 24 h. A single colony was then inoculated into 5 mL of LB broth and cultured overnight at 37 °C. Following incubation, 50 μL of overnight culture was used to inoculate 5 mL of LB and incubated at 37 °C with agitation until the optical density at 600 nm (OD600) reached 0.5. The cultures were then held on ice until ready for use.

### 2.2. Lytic Spectrum Determination

First, the ability of phage VR26 to infect different serotypes of STEC at 22 and 8 °C was determined using a quantitative spot dilution test as described in [32]. The host was classified as sensitive when single plaques could be detected. Then, determination of the efficiency of plating (EOP) was performed as described in [29]. In both experiments, the plates were incubated at room temperature (22 °C) for 24 h and at 8 °C for 5–6 days.

### 2.3. pH Tolerance of VR26

To determine the effect of pH on the stability of phage VR26, 10 μL of phage suspension (~11 log_10_ PFU/mL) was added to the tubes containing sterile PBS (90 μL) with pH values ranging from 2 to 13, adjusted with NaOH or HCl. The tubes were allowed to incubate at 22 °C for 60 min, then a double agar overlay plaque assay was performed [33]. Briefly, the phage solutions were serially diluted and plated in duplicate using STEC O26:H11 as a host. The plaques were counted, and the results were expressed as phage concentration (log_10_ PFU/mL).

### 2.4. Bacterial Challenge Assay

For the bacterial challenge assay, 5 mL of fresh bacterial culture, diluted to a final concentration of 4 log_10_ CFU/mL with LB broth, was inoculated with VR26 at ~8 log_10_ PFU/mL and incubated at 4 and 22 °C, at an agitation rate of 170 rpm. Culture without the addition of VR26 was used as a control. After 2, 4, 6, and 24 h of incubation, 100 μL of each sample was withdrawn, serially diluted in PBS buffer, if necessary, and plated on LB agar. The plates were incubated at 37 °C. Bacterial viable counts were determined after overnight incubation.

### 2.5. Treatment of Inoculated Fresh-Cut Lettuce with VR26

Iceberg lettuce (*Lactuca sativa*) was purchased at a grocery store IKI in Vilnius (Lithuania). Outer leaves of the lettuce were removed whereas the inner leaves were cut into pieces of ~1 by ~1 cm with a sterile, stainless steel scalpel. The samples were then transferred to the sterile Petri dishes and spot inoculated with 10 μL of STEC O26:H11 (4 log_10_ CFU/cm^2^). Inoculated lettuce was then treated with the phage VR26 suspension (8 log_10_ PFU/cm^2^) or PBS (control) and the samples were stored at 4, 8 and 22 °C. After 2, 4, 6, and 24 h of incubation, one sample per treatment was withdrawn, transferred to 2 ml Eppendorf tube with 1 mL PBS, and then homogenized with sterile bars and vortexed. Homogenates or serial dilutions of homogenates were plated on LB agar, and bacterial viable counts were determined after overnight incubation at 37 °C.

### 2.6. Statistical Analysis

All experiments were run in triplicate and bacteria/phage counts were determined by duplicate plating. Colony counts are presented as mean values with standard deviation. Unpaired *t*-test (two-tailed) was employed to calculate the statistical significance (*p* < 0.05) between treatments and control.

## 3. Results and Discussion

### 3.1. Lytic Spectrum and pH Tolerance of VR26

Bacteriophage VR26 was tested against a set of 5 pathogenic *E. coli*, representing different STEC serotypes: O157:H7, O103:H2, O111:H8, O145:H128, and O26:H11. A quantitative spot dilution test (Table 1) revealed that VR26 was capable of infecting only O26:H11 isolate, with roughly the same efficiency at both temperatures tested (8 and 22 °C).

Phages that display a narrow lytic activity against non-O157 *E. coli* were also described in a study by Litt et al. [24], in which three viruses targeting O26:H11 failed to infect isolates representing other STEC serogroups, including O45, O103, O111, O121, and O145. While it has been suggested previously that broad-spectrum phages are preferred candidates for a number of phage-based applications [23,24] phages with narrow host specificity could be used either to target a specific pathogen or in the development of phage cocktails with broad lytic activity against foodborne bacteria.

As seen in Table 1, VR26 had a slightly higher EOP on O26:H11 than it did on MH1. However, MH1 is a laboratory host which has been used for the isolation of bacteriophages, VR26 included, from the municipal wastewater samples [29]. Considering the origin of VR26, one could speculate that the natural host of this phage may just as well be a STEC strain.

**Figure 1:**
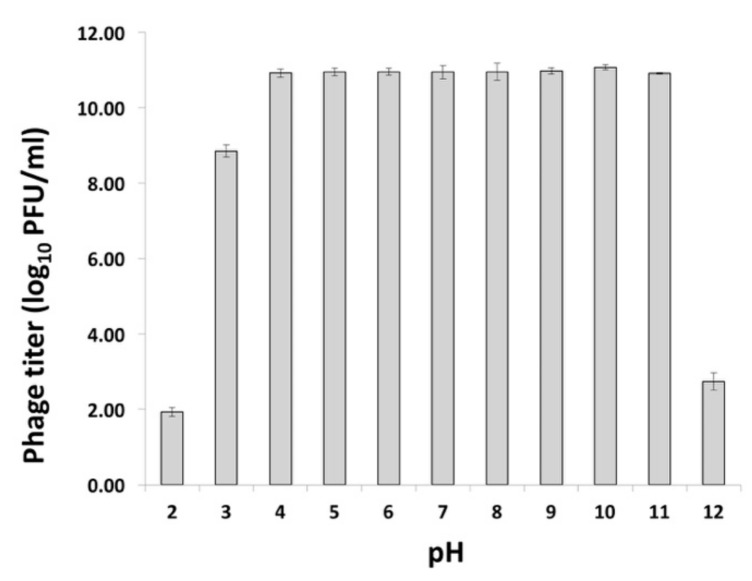
The effect of pH on VR26. Bars present standard deviation.

Since STEC strains are known to tolerate a somewhat broad range of pH (4.0–9.0) [34], bacteriophages intended for use in phage-based biocontrol must retain their lytic activity under similar conditions. To examine the effect of pH on VR26, the pH-inactivation assay (pH 2.0 to 12.0) was performed. As seen in Figure 1, VR26 was stable at pH values of 3–11, whereas significant reduction (8–9 log_10_ PFU/mL) in the viable titer was observed only after the exposure to extremely acidic (pH ≤ 2) or basic (pH ≥ 12) conditions.

Taken together, the results of the pH-inactivation assay suggested that VR26 sustained its lytic property through a broad range of pHs, indicating greater stability of this phage compared with other O26:H11-infecting [24] or even O157:H7-infecting viruses [24,35,36,37] described in previous studies.

The ability of phages to survive acidic (pH ≤ 5) or alkaline (pH ≥ 7) conditions is a desirable trait in phage-based applications. It has been suggested previously that acid-tolerant phages could be utilized to control pathogen colonization in the gastrointestinal tract (pH 1–5) of food animals if used as animal feed and/or water supplements [24,38,39,40]. Such phages could also be used as biocontrol agents in acidic foods such as fruit juices, jams, and fermented products [24]. Moreover, bacteriophages capable of surviving both acidic and alkaline pH could be used to address contamination by foodborne pathogens at a variety of time points during food production; applying to livestock animals before processing, decontaminating food preparation surfaces, or treatment of post-harvest food products [13,24].

### 3.2. Bacterial Challenge Assay

**Figure 2:**
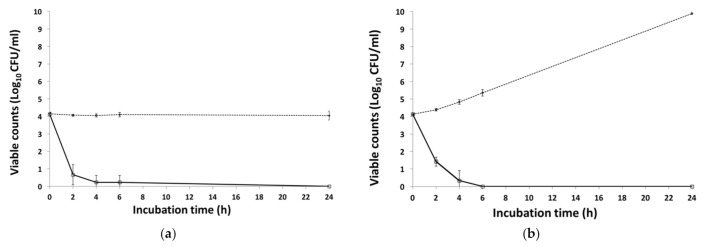
Effect of phage VR26 on numbers of STEC O26:H11 in LB medium at 4 (**a**) and 22 °C (**b**). Solid line, VR26-treated cultures; dashed line, control. Bars present standard deviation.

Compared with the phage-free controls, a significant reduction (*p* < 0.01) of O26:H11 in LB broth was observed after the addition of VR26, at both 22 °C and 4 °C (Figure 2).

After 2 h of incubation VR26 reduced O26:H11 counts by more than 2 log (*p* < 0.01) at 22 °C, and by approximately 3 log (*p* < 0.01) at 4 °C. VR26 was able to reduce (*p* < 0.01) numbers of O26:H11 to <10 CFU/mL after 4 h of incubation at 4 °C, whereas, after 24 h, the viable cells were not detected (Figure 2a). At 22 °C, O26:H11 was undetectable (<10 CFU/mL) after 4 h of phage treatment and no bacterial regrowth was observed thereafter (Figure 2b).

As discussed above, two reports describing phages capable of reducing O26:H11 levels in a broth culture system under standard laboratory conditions may be found in literature [27,28]. In a study by Niu et al. [27], a polyvalent T5virus vB_EcoS_AKFV33 was shown to decrease the viable counts of O26:H11 in broth culture by 7.5 ± 0.4 log_10_ CFU/mL (*p* < 0.001) at 37 °C but the efficacy of the phage (*p* < 0.001) was reduced after 7 h of incubation and regrowth of bacteria was seen thereafter. Mangieri et al. [28] reported that in 6 h at 37 °C a cocktail containing coliphages FM10, DP16, and DP19 caused a substantial reduction in at least 1 out of 10 STEC O26 serogroup strains tested. However, in the latter study, viable cell enumeration was not performed and the measurement of optical density (OD) at 600 nm was used instead. Also, since the H-antigen has not been specified by the authors, it is unclear how many of the aforementioned strains are of O26:H11 serotype.

The results presented here are hardly comparable with those obtained during the aforementioned studies since bacterial challenge with phage VR26 assay was performed at 4 and 22 °C, not at 37 °C. However, in the food industry, foods are hardly ever exposed to 37 °C. Hence, the ability of VR26 to effectively reduce O26:H11 at both refrigeration and room temperature conditions indicates that this bacterial virus shows potential in the decontamination of foods, especially at post-harvest.

### 3.3. Effect of VR26 on O26:H11 on Fresh Lettuce

**Figure 3:**
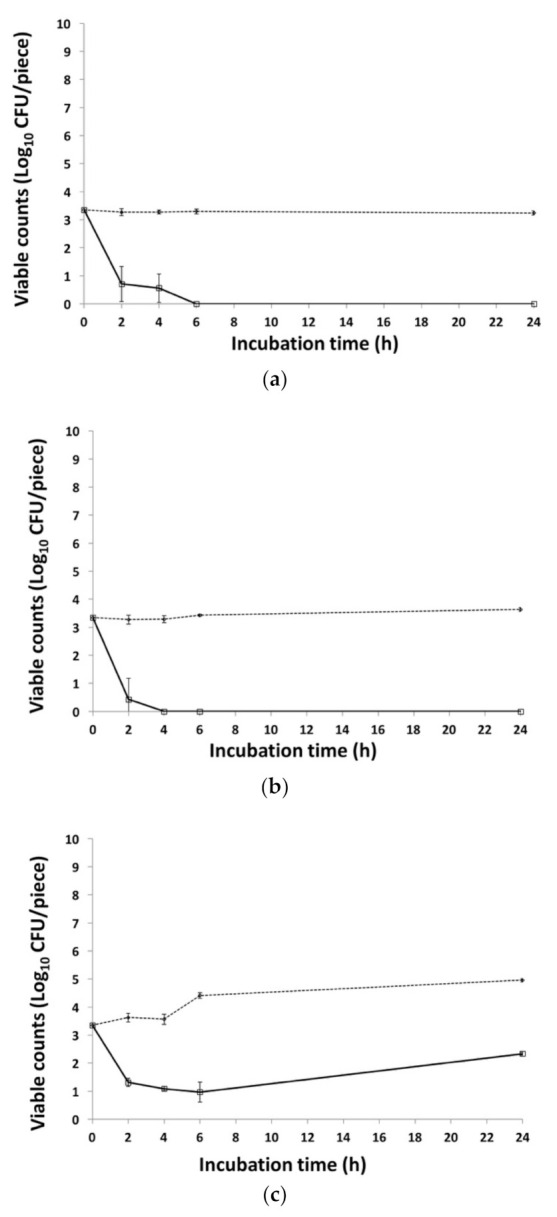
Effect of phage VR26 on numbers of STEC O26:H11 on fresh lettuce at 4 (**a**), 8 (**b**), and 22 °C (**c**). Solid line, VR26-treated lettuce pieces; dashed line, control. Bars present standard deviation.

The ability of the phage VR26 to reduce O26:H11 on lettuce was examined at 4, 8, and 22 °C. As seen in Figure 3, VR26 reduced (*p* < 0.02) the viable counts of O26:H11 by ~2 log_10_ CFU/piece after 2 h of treatment at all temperatures. No survivors were detected after 6 and 4 h of treatment at 4 and 8 °C, respectively.

At 22 °C, the pathogen was reduced by more than 2 log_10_ CFU within 6 h and, although bacterial regrowth was observed afterward, O26:H11 counts on non-treated lettuce pieces were >2 log_10_ CFU/piece higher than on phage-treated samples (2.3 ± 0.06 log_10_ CFU/piece) (*p* < 0.02).

Bacterial regrowth observed during phage treatment at room temperature was also reported by a number of previous studies [17,41,42,43,44] and could be explained either by the inability of viral particles to “move” and, consequently, to find all host-cells on solid foods or by the appearance of phage-resistant bacteria [17,45,46]. However, the probability of phage and cell coming into contact could be increased by applying higher phage concentrations, whereas, to prevent the emergence of phage resistance, so-called “phage cocktails” or phages in combination with other antibacterial agents could be used [17,47,48,49,50]. Moreover, in the modern food industry, the probability of foods being contaminated with high numbers of pathogenic microorganisms is relatively low [48] which, in itself, minimizes the occurrence of phage resistance.

## 4. Conclusions

Here, for the first time, the efficacy of low-temperature *E. coli* virus VR26 in the biocontrol of foodborne pathogens was evaluated. Our results clearly demonstrate that bacteriophage VR26 retained its lytic activity after being exposed to a range of pH from 3 to 11 and efficiently reduced O26:H11 counts, both in broth culture and on fresh lettuce, at all temperatures tested. Moreover, the greatest efficacy of VR26 on food was observed at common refrigeration temperatures (4 and 8 °C), suggesting that this phage could potentially be used as a biocontrol agent against STEC O26:H11 in the food industry.

## Figures and Tables

**Table 1:** Susceptibility of STEC isolates to VR26.

Strain/Serotype	Spot Test		EOP	
	8 °C	22 °C	8 °C	22 °C
O26:H11	+	+	0.89	1.0
O157:H7	-	-	-	-
O103:H2	-	-	-	-
O111:H8	-	-	-	-
O145:H128	-	-	-	-
MH1 ^1^	+	+	0.84	0.99

^1^ host strain; “+”—clear plaques were observed; “-“—no plaques; 1—the largest number of plaques (taken as a standard in EOP calculation).

## Data Availability

The complete genome sequence of *E. coli* phage VR26 is available at the NCBI, accession number NC_028957.
